# Bioactivities, Applications, Safety, and Health Benefits of Bioactive Peptides From Food and By-Products: A Review

**DOI:** 10.3389/fnut.2021.815640

**Published:** 2022-01-20

**Authors:** Ahmed A. Zaky, Jesus Simal-Gandara, Jong-Bang Eun, Jae-Han Shim, A. M. Abd El-Aty

**Affiliations:** ^1^National Research Centre, Department of Food Technology, Food Industries and Nutrition Research Institute, Cairo, Egypt; ^2^Nutrition and Bromatology Group, Department of Analytical Chemistry and Food Science, Faculty of Science, Universidade de Vigo, Ourense, Spain; ^3^Department of Food Science and Technology, Chonnam National University, Gwangju, South Korea; ^4^Natural Products Chemistry Laboratory, Biotechnology Research Institute, Chonnam National University, Gwangju, South Korea; ^5^Department of Pharmacology, Faculty of Veterinary Medicine, Cairo University, Giza, Egypt; ^6^Department of Medical Pharmacology, Medical Faculty, Ataturk University, Erzurum, Turkey

**Keywords:** bioactive peptides, health benefits, bioactivities, applications, safety

## Abstract

Bioactive peptides generated from food proteins have great potential as functional foods and nutraceuticals. Bioactive peptides possess several significant functions, such as antioxidative, anti-inflammatory, anticancer, antimicrobial, immunomodulatory, and antihypertensive effects in the living body. In recent years, numerous reports have been published describing bioactive peptides/hydrolysates produced from various food sources. Herein, we reviewed the bioactive peptides or protein hydrolysates found in the plant, animal, marine, and dairy products, as well as their by-products. This review also emphasizes the health benefits, bioactivities, and utilization of active peptides obtained from the mentioned sources. Their possible application in functional product development, feed, wound healing, pharmaceutical and cosmetic industries, and their use as food additives have all been investigated alongside considerations on their safety.

## Introduction

Nowadays, food is recognized as a source of dietary substances and biologically active compounds that improve human health and the general conditions of the organism. The consumers' increasing awareness of the influence of diet on health is reflected in their selection of natural products, abundant in vitamins, minerals, and other bioactive compounds like carotenoids (1), anthocyanins (2), polyphenols (3), or peptides (4, 5).

Bioactive peptides are protein fragments that benefit the body systems and overall human health. Most bioactive peptides range between two (dipeptides) and 20 amino acid residues and have a molecular mass of 0.4–2 kDa (6). Longer peptides have also been reported in rare cases. Lunasin, for example, is a peptide formed by 43 amino acids produced from soy, which demonstrates anti-cancer and hypocholesterolemic properties (7).

Bioactive peptides generated from food possess an excellent potential for creating functional foods and/or nutraceuticals to prevent or treat some chronic diseases (8). Many articles on the generation and characterization of bioactive peptides with antimicrobial, anti-inflammatory, antihypertensive, anti-obesity, and antioxidant attributes have been published (9). Herein, we focused on bioactive peptides from different foods and their by-products, their effects on health, and possible applications.

In this investigation, eligible studies (in English) were acknowledged during an electronic search of the PubMed database (1991–2021) (https://www.nlm.nih.gov/) and Google. We employed the chief search word “bioactive peptides” along with the words “sources,” “by-products,” “extraction,” “purification,” “identification,” “bioactivities,” “health effects,” “pharmaceutical applications,” “food applications,” “cosmeceutical applications,” “feed applications,” and “safety” to find the relevant articles. We selected the titles, keywords, and abstracts of the articles collected from the database. Several review articles were omitted in favor of the primary sources cited.

## The Sources of Bioactive Peptides

Peptides and proteins are critical macronutrients as they provide the necessary raw materials for protein production and serve as a source of energy. Bioactive peptides have been isolated or produced from various plant and animal sources (Tables 1–4). Food proteins are chosen as a reference for bioactive peptides based on two factors: (i) a desire to add value to abundant underused proteins or protein-rich industrial food waste, and (ii) the use of proteins with particular peptide sequences or amino acid residues with specific pharmacological benefits (87).

**Table 1:** Peptides from milk and by-products and their bioactivity.

**Source**	**Peptide sequence**	**Bioactivity**	**References**
Bovine milk	Lys-Val-Leu-Pro-Val-P(Glu)	Antihypertensive activity	(10)
	N/A	Antimicrobial activity	(11)
Cheddar cheeses	N/A	Antimicrobial, antioxidant, and antihypertensive activity	(12)
	N/A	Phosphopeptides	(13)
Casein hydrolysates	Arg-Tyr-Lue-Gly-Tyr	Antihypertensive activity	(14)
Comte cheese	N/A	Phosphopeptides	(15)
Feta, Swiss cheeses	N/A	Antiamnesic	(16)
Mik fermented	Ile-Pro-Pro Val-Pro-Pro	ACE-inhibitory activity	(17)
Enzyme modified cheese	N/A	Opioid activity and ACE-inhibitory activity	(18)
Yogurt	N/A	Antihypertensive and antimicrobial activity	(19)
	κ-casein: Met-Ala-Ile	Antithrombotic activity	(20)
	N/A	Immunomodulatory	(13)
	N/A	Antithrombotic	(21)
β- lactoglobulin	β-lactosin B	Antihypertensive activity	(22)
		Antithrombotic activity	(23)
Fermented milk	Val-Pro-Pro,Ile-Pro-Pro	Antihypertensive activity	(24)
Sour milk	N/A	Phosphopeptides	(25)
	N/A	Antihypertensive properties	(16)
**By-products**			
Whey protein hydrolysate	Lactoferricin	Antimicrobial activity, anti-cancer	(24)
	N/A	Opioid activity	(26)
	TTFHTSGY GYDTQAIVQ	Antihypertensive properties	(13)
Whey proteins	N/A	Anticancer	(15)

*N/A, Not available*.

**Table 2:** Peptides from meat and by-products and their bioactivity.

**Source**	**Peptide sequence**	**Bioactivity**	**References**
Duck breast meat	LQAEVEELRAALE IEDPFDQDDWGAWKK	Antioxidant activity	(27)
Beef muscle	DFHING	Antihypertensive activity	(28)
Bovine brain	MPPPLPARVDFSLAGALN	Phosphoenolpyruvate inhibitory activity	(29)
Venison muscle	MQIFVKTLTG DLSDGEQGVL	Antioxidant activity	(30)
Beef muscle	GFHI, DFHING, FHG, GLSDGEWQ	Antimicrobial activity	(28)
Fermented meat sauce	GYP	Antioxidant activity	(31)
Chicken breast protein	Breast protein hydrolysate	Antioxidant activity	(32)
Bovine muscle	YEDCTDCGN	Anti-opioid activity and ACE-inhibitory activity	(29)
**By-products**			
Duck skin	N/A	Antioxidant activity	(33)
Bovine myoglobin	AKHPSDFGADAQA		(34)
Bovine blood	YPWT	Opioid activity	(29)
Bovine blood	TKAVEHLDDLPGALSELSDLHAHKLR VDPVNFKLLSHSLL	Antihypertensive activity	(35)
Bovine tendon	AKGANGAPGIAGAPGFPG ARGPSGPQGPSGPP		(36)
Bovine blood	STVLTSKYR	Antimicrobial activity	(37)
Buffalo horn	AADNANELFPPN	Antioxidant activity	(38)
Bovine skin	N/A		(39)
Bovine brain	N/A		(40)
Buffalo horn	AADNANELFPPN		(38)
Yak skin	<3 kDa		(41)
Sheep abomasum protein	LEDGLK		(42)
Bovine liver	<10 kDa		(43)
Dry-cured ham bones	N/A		(44)
Chicken liver	N/A		(45)
Chicken bone collagen hydrolysates	N/A	Lipid-lowering activity	(46)

*N/A, Not available*.

**Table 3:** Peptides from plants and by-products and their bioactivity.

**Source**	**Peptide sequence**	**Bioactivity**	**References**
Maize	RSGRGECRRQCLRRHEGQPWET QECMRRC RRR/ YA, LMCH (zein)/ LPP (zein)	Antimicrobial, antioxidant, and antihypertensive activities	(24)
Soybean	1–3 kDa	Antimicrobial activity	(47)
Oat and wheat grains	N/A	Antihypertensive, antioxidant, antithrombotic, and opioid activities	(48)
Sweet potato	N/A	Antioxidant activity	(49)
Corn protein	Pro-Phe and Leu-Pro- Phe		(50)
Amaranth	VW, GQ/PYY, RWY, WY, RW PWW, PWR, PW, PWY WYS/VGECVRGRCPSGMCCSQF GYCGKGPKYCG	Anticancer, antioxidant and antimicrobial activities	(24, 51)
Rice protein	Thr-Gln-Val-Tyr	ACE-inhibitory, antimicrobial, and antioxidant activities	(48, 52)
Lentils	N/A	Antioxidant activity	(53)
Zein hydrolysate	N/A	Antioxidant activity	(54)
Quinoa flour	5 peptides; <1.1 kDa		(55)
Moringa seed	Peptied fractions <10 kDa	Antidiabetic, antioxidant, and antidiabetic activities	(56)
**By-products**			
Plum by-product	N/A	Antioxidant and ACE inhibiting activities	(57)
Rice bran protein hydrolysates	N/A	Antioxidant activity	(4, 58)
Soybean meal	Peptied fractions: <5 kDa, 3–5 kDa, 1–3 kDa, >1 kDa	Antioxidant, antimicrobial, and antitumor activities	(59, 60)
Palm kernel oil cake	YLLLK YGIKVGYAIP GGIF GIFE GVQEGAGHYALL LPWRPATNVF	Antihypertensive activity	(61)
Wheat bran protein hydrolysates	Gluten	Antihypertensive and antioxidant activities	(62)
Sunflower seed meal	FVNPQAGS	Antihypertensive activity	(63)
Watermelon seed	Hydrophobic amino acids (Gly,Ala, Val, Met, Ile) Aromatic amino acids (Tyr, Phe, His)	Antioxidant activity	(64)
Tomato seed cake	10 peptides; <1 kDa	ACE inhibitory and antioxidant activities	(65)
Cottonseed meal	<1 kDa	Antioxidant activity	(66)
Corn gluten meal	Peptides fraction of 500–1,500 Da		(67)
Sesame meal	N/A		(68)

*N/A, Not available*.

**Table 4:** Peptides from marine and by-products and their bioactivity.

**Source**		**Bioactivity**	**References**
Shrimp proteins	N/A	Antihypertensive activity	(69)
Tuna proteins	N/A		(70)
Sea cucumber hydrolysate			(71)
Conger eel protein	LGLNGDDVN	Antioxidant activity	(72)
Sardine protein	LQPGQGQQ		(73)
Mackerel filet protein hydrolysate	N/A		(74)
Royal jelly protein	AL, FK, FR, IR, KF, KL, KY, RY, YD, YY, LDR, KNYP		(75)
Mollusks (*Conus magus*)	N/A	Analgesic	(24)
Seaweed (*Eucheuma serra*)	Lectins	Anticancer	(76)
Sponges (*Jaspis* spp.)	Jaspamide		(77)
*Geodia corticostylifera*	N/A	Antiproliferative	(78)
**By-products**			
Tilapia (*O. niloticus*) skin	Leu-Ser-Gly-Tyr-Gly-Pro	Antihypertensive activity	Chen et al. (79)
Pacific cod skin gelatin	N/A		(80)
Tuna backbone	VKAGFAWTANQQLS	Antioxidant activity	(81)
Hoki skin gelatin	HGPLGPL		(82)
Salmon (Protamine, derived from fish milt)	Pro-Arg (271.3 Da)		(83)
Horse mackerel viscera	Ala-Cys-Phe-Leu		(84)
Olive flounder (*P*. *olivaceus*) surimi	N/A	Antihypertensive activity	(85)
Bluefin leatherjacket heads	Trp-Glu-Gly-ProLys; Gly-Pro-Pro; Gly-Val-Pro-Leu-Thr	Antioxidant activity	(86)

*N/A, Not available*.

### Extraction of Bioactive Peptides

**Figure 1:**
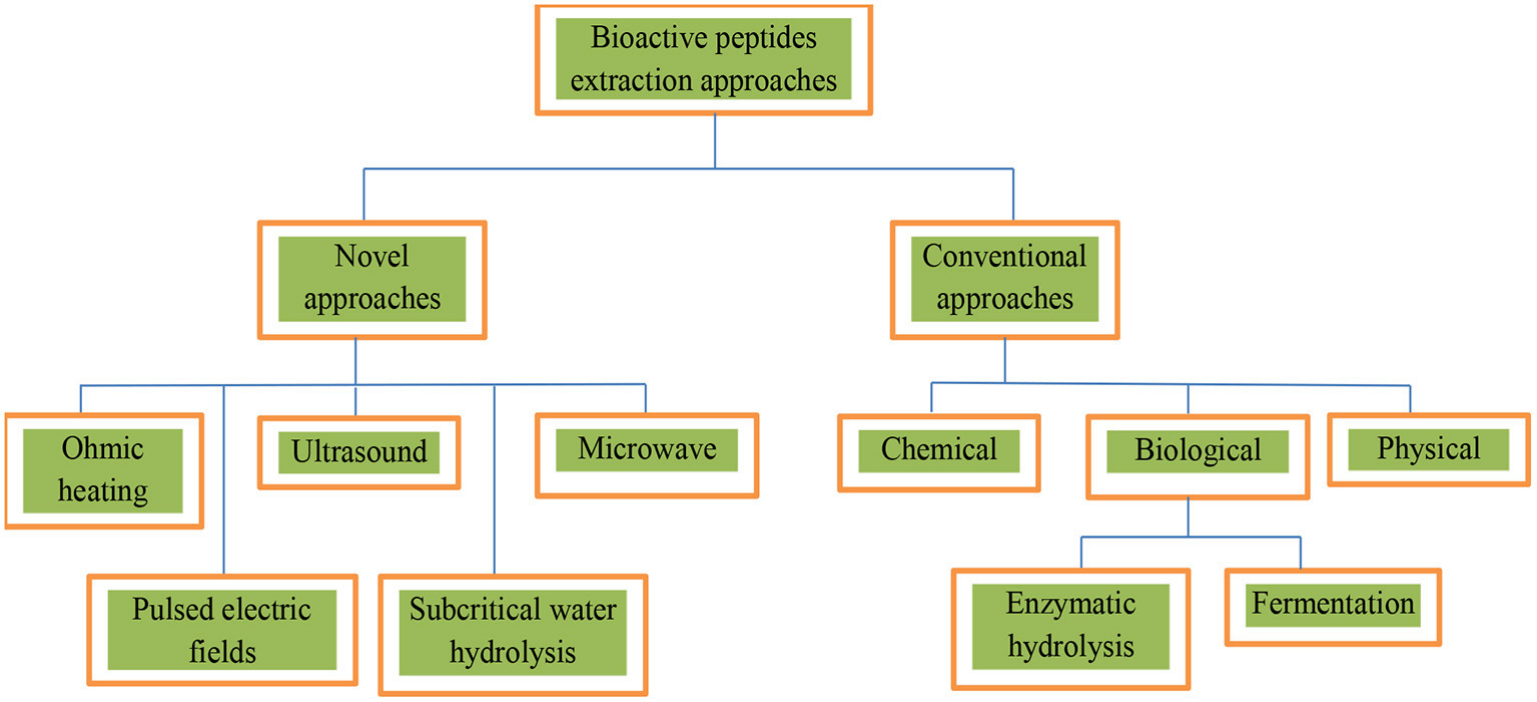
Scheme for extracting bioactive peptides.

Bioactive peptides are conventionally isolated by chemical or enzymatic hydrolysis and fermentation. To enhance the degree of hydrolysis in the generation of bioactive peptides, new approaches, such as microwave, ultrasound-assisted extraction, ohmic heating, pulsed electric fields, and subcritical water hydrolysis, have been investigated (88). Physical processes are at the core of these techniques (Figure 1).

### Chemical Methods

Chemical techniques using alkalis, such as sodium hydroxide, are the most typical and conventional method for protein extraction from plant sources (89, 90). It can effectively break hydrogen and amide bonds to solubilize rice bran proteins. Although this process is highly effective in obtaining most proteins in a soluble form, it creates specific structural changes that cause a protein to lose its original function (91).

### Enzymatic Methods

Enzymatic hydrolysis is another common approach for separating proteins and hydrolysates/peptides from various food sources (92). Enzymes are employed in diverse ways to facilitate protein extraction from food, such as cell wall degradation, starch-bond protein release, and protein solubility improvement (93). In this regard, Wang et al. (94) utilized phytase and xylanase to isolate protein from rice bran and noticed that the use of carbohydrates could be helpful to improve the yield of soluble protein.

### Physical Methods

Physical methods are often favored over chemical or enzymatic treatments for food production because they have fewer changes (95). These techniques are more economical and easy to adapt and use in the industry. Conventional physical procedures, such as colloidal milling, homogenization, high-speed blending, freeze-thaw, and high pressure, have been utilized for protein extraction (90).

### Microwave-Assisted Extraction

Microwave heating is a novel technology based on electromagnetic waves with wavelengths and frequencies ranging from 1 mm to 1 m and 300 MHz to 300 GHz, respectively. It has gained popularity in the food processing industry because of its uniform heating, high heating rates, safety, simple, quick, and clean operation, and low maintenance. Furthermore, this kind of heating has a lower impact on food products' flavor and nutritional quality than conventional heating. By shattering disulfide and hydrogen bonds (non-covalent bonds), this approach can cause protein unfolding, which affects the secondary and tertiary structures of proteins (96, 97). In this respect, the microwave process was shown to assist the chia seed protein enzymatic hydrolysis with enhanced bioactivity (antioxidant activity), and functionality (emulsification and foaming properties) gained in a shorter time in comparison to traditional hydrolysis techniques (98).

### Ultrasound-Assisted Extraction

Sonication is a green, novel, innovative and sustainable strategy based on high sound waves of frequencies (>16 kHz) undetectable by the human ear. This approach has several benefits compared with traditional thermal processes, including higher efficiency, higher rate, more accessible and cheaper application and operation, lower equipment contamination, and higher quality and functionality of processed foods (98, 99). In this context, Zhao et al. (100) demonstrated that sonication with power levels of 200, 400, or 600 W for 15 or 30 min altered the secondary and tertiary structure of walnut protein isolate without any impact on its primary structure since the process could not break the covalent bonds. Further, Vanga et al. (101) indicated that ultrasonic treatment (25 kHz, 400 W, 1–16 min) reduced soymilk protein trypsin inhibitor activity by 52% and enhanced its digestibility.

### Ohmic Heating

Ohmic heating is a thermal processing technology that applies alternating electric currents directly into a semi-conductive media. It was initially employed for milk pasteurization in 1920. According to Joule's law, direct or volumetric heat is generated in products by passing a moderate and alternating electric current through them, which functions as resistance in an electrical circuit (102, 103). In this way, Li et al. (104) evaluated the structure and techno-functionality of proteins in soybean milk when using ohmic heating against traditional heating. Their findings revealed that ohmic heating effectively reduced heating time and enhanced the protein's emulsifying capacity. The protein's foaming ability, on the other hand, reduced as its surface hydrophobicity dropped.

### The Pulsed-Electric Field (PEF)

The pulsed-electric field (PEF) technique has been employed as a non-thermal process for microorganisms and enzymes inactivation. In this technology, the food sample is subjected to short high-power electrical pulses (μs or ms) between electrodes (105). A PEF system consists of a chamber, electrodes, a high-voltage pulse generator, and a computer for monitoring and controlling devices. A strong electric field is formed between two electrodes because of their electrical potential difference. During the PEF process, the generated electrical energy might cause protein unfolding and enhanced interactions with the solute. This can impact the peptides/protein's functional characteristics by increasing its solubility (106). In this regard, PEF treatment of canola seeds enhanced the extracted protein's solubility, emulsifying, and foaming capabilities, according to Zhang et al. (107). Nevertheless, depending on the strength and duration of the PEF process, it can result in denaturation and aggregation, resulting in decreased solubility. The PEF method can change plant-derived peptides and proteins' secondary and tertiary structures. Changes in the secondary structure of peptides derived from pine nut protein were also informed, along with their antioxidant effect (108).

### Purification and Identification of Bioactive Compounds

All the methods for purifying and identifying bioactive peptides are very similar. Purification of active peptides is required to produce a commercially viable product. Ultrafiltration, RP-HPLC, size exclusion chromatography, and ion-exchange chromatography, can all be used to purify bioactive peptides. Additionally, for protein identification, analytical techniques such as mass spectrometry (MS), electrospray ionization MS, matrix-assisted laser desorption ionization time-of-flight MS, liquid chromatography-MS/MS, and hydrophilic interaction liquid chromatography (HILIC) are widely utilized (109).

## Bioactivities of Bioactive Peptides and Their Impact on Health

**Figure 2:**
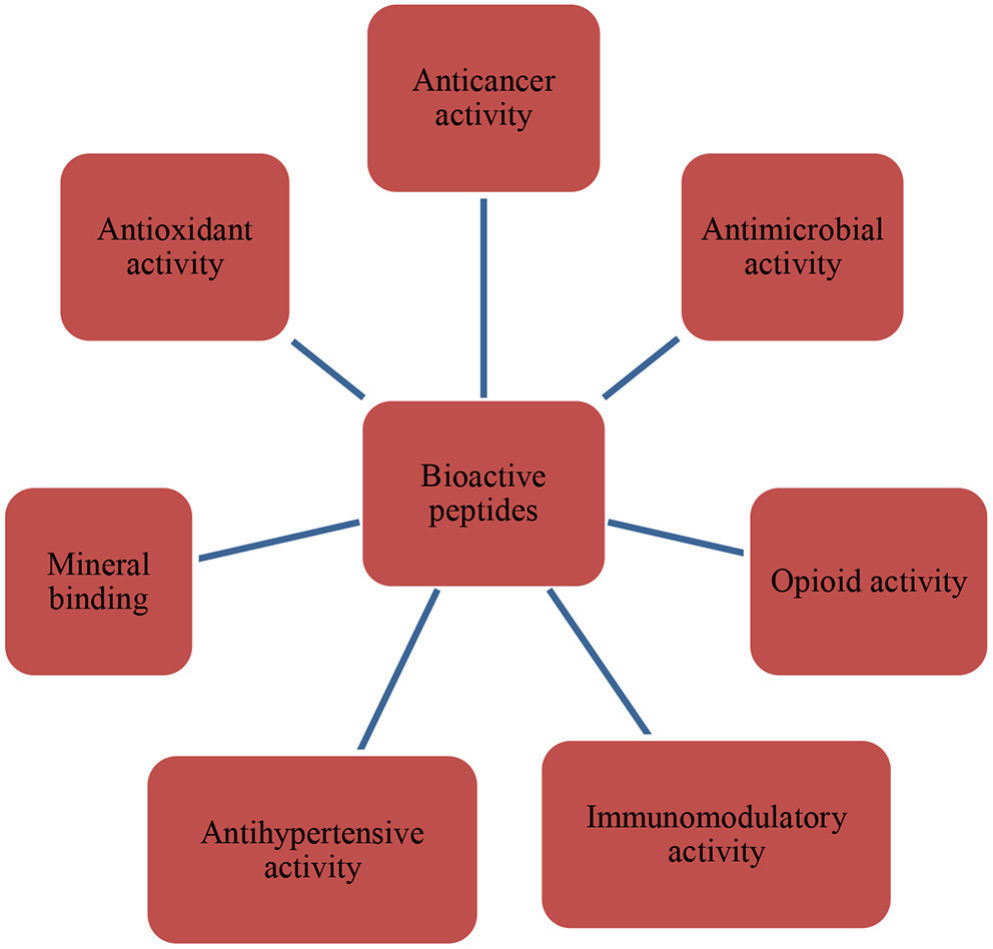
Bioactivities of bioactive peptides.

Proteins are necessary for the growth and the preservation of many biological processes. The awareness regarding physiologically active peptides is growing quickly, as they may serve as possible modifiers for several regulative functions in the body. Bioactive peptides have different biological actions depending on the amino acid class, net charge, secondary structures, sequence, and molecular mass (110). Multiple studies have determined the bioactivities of peptides, which were linked to improved overall health and a lower risk of specific chronic diseases, such as cancer, diabetes, and heart diseases (Figure 2).

### Antioxidant Activity

Reactive oxygen species cause cell damage, leading to cancer, diabetes, cardiovascular disease, and hypertension (111). The antioxidative characteristics of bioactive peptides are associated with their composition, formation, and hydrophobicity. Histidine, glutamic acid, proline, tyrosine, cysteine, methionine, and phenylalanine are all amino acids with antioxidant properties (112). Amino acids bind pro-oxidant metal ions to perform their activity, scavenge the OH radical and/or inhibit lipid peroxidation. As a result, each amino acid contributes as an antioxidant uniquely, depending on its type (67). Most antioxidant peptides include 4–16 amino acid residues and have a molecular mass of 0.4–2 kDa. Peptide molecular size influences both the pathways to target locations and the gastrointestinal digesting process, potentially increasing antioxidant activity *in vivo* (113). Tyrosine-containing peptides work primarily through hydrogen atom transfer, whereas cysteine, tryptophan, and histidine-containing peptides work mainly through single electron transfer (114). Aromatic amino acids like Tyr and Phe are excellent at donating protons to electron-deficient radicals. This characteristic enhances the bioactive peptides' radical-scavenging abilities. The antioxidant capacity of His-containing peptides is confirmed to be linked to hydrogen donating and lipid peroxyl radical trapping (115). The sulfhydryl group in cysteines, on the other hand, is endowed with an antioxidant effect because of its primary reaction with radicals (116). Plant-based proteins derived from industrial food and its by-products, such as soybean, wheat germ, hemp seeds, rice bran, sesame bran, wheat bran, and rapeseed, possess bioactive peptides with antioxidant characteristics (117).

### Antimicrobial Activity

Antimicrobial peptides possess an antimicrobial activity that protects mammals from various bacteria, fungi, and viruses. Antimicrobial activity is also a coveted feature in prepared foods since it directly impacts the product's shelf life. Antimicrobial peptides are divided into three categories: short (20–46 amino acid residues), basic (rich in Lys or Are), and amphipathic. They are commonly abundant in hydrophobic residues, such as Leu, Ile, Val, Phe, and Try (118). Multicellular organisms create antimicrobial peptides as defensive strategies against pathogenic microorganisms. Antimicrobial peptides can alter the cell membrane and biological processes, including cell division (119). Their action is assumed to create channels or pores within bacterial membranes, inhibiting anabolic activities, changes in gene expression and signaling transduction, and promoting angiogenesis. For example, the antimicrobial action of milk is demonstrated by extensive research. Lactoferrin, which is hydrolyzed into lactoferricin in the gastrointestinal tract, is an essential contributor to the synthesis of various other bioactive peptides and has antimicrobial ability in and of itself (120). Antimicrobial peptides have also been discovered in marine products. Many microorganisms, like *Staphylococcus aureus, Escherichia coli, Bacillus subtilis, Shigella dysenteriae, Pseudomonas aeruginosa, Salmonella typhimurium*, and *Streptococcus pneumoniae*, were inhibited by the peptide GLSRLFTALK, isolated from anchovy cooking wastewater (121). Moreover, Aguilar-Toalá et al. (122) found that adding chia protein hydrolysate (<3 kDa) possessed higher antimicrobial activity than both chia peptide fraction 3–10 kDa. Furthermore, the <3 kDa fraction demonstrated a notable increase in membrane permeability of *E. coli* (71.49% crystal violet uptake) and *L. monocytogenes* (80.10% crystal violet uptake).

### Mineral Binding

At intestinal pH, peptides with specific sequences create compounds by binding in solution with minerals, such as calcium (Ca) and phosphorus (P). As these peptides have a higher anionic character, they form soluble complexes immune to additional proteolytic attacks, blocking the creation of insoluble mineral compounds (24). Flaxseed proteins contain hydrophobic and positively charged amino acids that might aid enzymatic hydrolysis in generating calmodulin (CaM)-binding peptides. Flaxseed proteins were digested with alcalase to produce low-MW peptides (123). Milk caseins are also known to bind Ca and P ions, increasing their bioavailability (24).

### Opioid Activity

Opioid peptides are naturally synthesized and have analgesic properties (124). They bind to the opiate receptor and exhibit opiate-like actions suppressed by naloxone (125), with a specific impact on the neurological system (126). Prodynorphin (dynorphins), proopiomelanocortin (endorphins), and proenkephalin (enkephalin) are the three types of precursor proteins found in typical opioid peptides (127). The N-terminal sequences Tyr-Gly-Gly-Phe and Tyr-Pro are prevalent in these peptides (113). Opioid peptides can be found in milk and dairy products (18) and various plant sources, including oats, wheat, rye, barley, and maize (128). Pihlanto-Leppälä (129) found that opioid peptides interact with particular receptors on target cells.

### Anticancer Activity

Cancer has become one of the world's most feared and deadly diseases. Pharmaceutical companies are developing anticancer and antitumor medications at a rapid pace. Further, oncology research is well-progressed and has improved our understanding of tumors over time (130). Food protein hydrolysate is an excellent foundation for the generation of anticancer peptides. The anticancer effect of rice and soy protein hydrolysates has been previously demonstrated. In rice, anticancer peptides are produced by alcalase digestion of rice bran proteins (131). Another anticancer peptide (Ala-Phe-Asn-Ile-His-Asn-Arg-Asn-Leu-Leu) was separated from shellfish proteins, which successfully killed breast, prostate, and lung cancer cells while leaving normal liver cells unharmed (132). Most anticancer investigations on peptides are conducted on lunasin; a peptide derived from soy or wheat grains (24). The anticancer properties of lunasin are linked to its particular amino acid sequences, which contain Arg-Gly-Asp for cell adhesion and a polyaspartic acid chain with nine aspartic acid residues (133). Fermented soybean extracts impact the proliferation of MCF7 breast cancer cells and downregulate gene expression, according to Hwang et al. (134). The investigators found that, through stimulating the TGF pathway, fermented soybean extracts may effectively prevent breast cancer. According to Badger et al. (135), soy peptide concentrates reduce the incidence of breast, prostate, and gastrointestinal cancers. They claimed that soy peptide concentrates could reduce cancer incidence by 80%. Further, peptides from black soybean, mung bean meal, and adzuki bean were found to suppress cancer cells at 200–600 g/mL concentrations (136). These anticancer peptides were only studied *in vitro*; further investigation on their bioavailability is needed.

### Antihypertensive Activity

Owing to the changes in lifestyle in modern society, there is a growing need for functional foods with blood-pressure-lowering benefits in the therapy of hypertension. Hypertension can cause multiple disorders, including heart and renal diseases, arteriosclerosis, and stroke (137). Antihypertensive peptides (also known as angiotensin-converting enzyme (ACE) inhibitors) generated by protein hydrolysates are the most studied peptides (138). In this respect, ACE has a crucial effect since it catalyzes the transformation of angiotensin I to angiotensin II, which leads to a rise in blood pressure. Aromatic amino acid residues at the C-terminus and hydrophobic amino acid residues at the N-terminus help peptides block ACE function more effectively (139). Various plant sources, including pea (Ile-Arg, Lys-Phe, and Glu-Phe), soybean (Asp-Leu-Pro and Asp-Gly), and rice (Ile-His-Arg-Phe), have been shown to possess active peptides with antihypertensive capacity (117). Marambe et al. (140) found that defatted flaxseed protein hydrolysate reduced the ACE activity, lowering the risk of cardiovascular disorders. Many tripeptides that restrain ACE have been separated from foods. In this context, Wang et al. (141) confirmed that an active peptide (Tyr-Ser-Lys) derived from rice bran had a potent ACE inhibitory effect. Another work by Tuomilehto et al. (17) found that the milk-obtained bioactive tripeptides (Val-Pro-Pro and Ile-Pro-Pro) lowered blood pressure in moderately hypertensive patients. Bioactive peptides, particularly those with low molecular weight, inhibited ACE, decreased blood pressure and prevented hypertension.

### Immunomodulatory Activity

Immunomodulatory activity is essential for the human immune system to function correctly. The immunomodulatory effect of bioactive peptides depends on cytokine regulation, antibody formation, immune system stimulation *via* reactive oxygen species, conformational changes in tubulin, and inhibition of protein synthesis (87). Furthermore, the amino acid content, sequence, length, charge, hydrophobicity, and peptide structure are linked to the immunomodulatory function. In this regard, soy protein hydrolysates with low molecular weight and many positively charged peptides have been proven to stimulate immunomodulation (142). Numerous plant-generated bioactive peptides with immunomodulatory action, including Leu-Asp-Ala-Val-Asn-Arg and Met-Met-Leu-Asp-Phe, possess low molecular weights (686 and 655 Da, respectively) and hydrophobic characteristics (143). According to Ngo et al. (144), marine products are a significant source of bioactive peptides that have been used as a treatment for a variety of disorders.

### Anti-inflammatory Activity

Anti-inflammatory effects have been found in proteins/peptides derived from eggs, milk, and plants (145). The anti-inflammatory characteristics of new active peptides from sponges, bacteria, and microalgae have been documented, along with the molecular diversity of marine peptides and data regarding their anti-inflammatory impact and modes of action (146). Zhao et al. (147) reported that anti-inflammatory peptides generated from velvet antler simulated gastrointestinal digests were purified and identified using LC-MS/MS. Four anti-inflammatory peptides were identified, namely VH, LAN, AL, and IA. These findings proposed that peptides obtained from velvet antler protein might be a viable anti-inflammatory agent in functional ingredients. Bioactive peptides promoted diet-induced hepatic fat deposition and hepatocyte pro-inflammatory response when evaluated on SAMP8 aging rats (148). *In vitro* and *in vivo* investigations have revealed that corn, whey, and soybean protein hydrolysates have a powerful anti-inflammatory effect (149, 150).

## Applications of Bioactive Peptides

### Food Applications

Bioactive peptides have shown to be extremely useful in developing numerous health-oriented functional diets. These peptides are used as sweeteners, color stabilizers, thickeners, anti-caking factors, emulsifiers, flavor enhancers, emulsifiers in food preparation, and acidity control. Bioactive peptides may also improve food quality by affecting the water and oil retention capacity, colloidal stability, viscosity, and foam generation in the finished product (151). Peptide isolates used in the formulation of functional products aid in creating certain required technical qualities. Numerous studies have been conducted on proteins/peptides of different origins to produce functional foods. Emulsification is a necessary procedure that is frequently utilized to assess protein-rich products. Due to their amphiphilic character, bioactive peptides derived from food by-products are important for emulsifying attributes (152). Active peptides derived from plant sources like potato (153), flaxseed (154), and soybean (155) have been found to exhibit emulsifying capabilities. As reported by Álvarez et al. (156), adding rice bran protein concentrate to beef products increased its emulsion stability and rheological qualities. Due to the increased levels of bioactive peptides, Talukder and Sharma (157) found that using oat bran concentrate in the formulation of chicken meat patties resulted in better emulsion activity than that obtained using wheat bran concentrate. Likewise, Kamani et al. (158) observed that soy protein concentrate and gluten in sausage recipes increased emulsion stability and gel-forming capabilities by producing a robust structural network.

Foam formation can generate acceptable textural and sensory characteristics in food such as pastries and sauces (159). Their capacity to reduce surface tension facilitates the use of active peptides as foam stabilizers. Rice bran protein isolates had a comparable foaming potential to egg white but much lower foaming stability (114). Similarly, Elsohemy et al. (160) reported that the foaming capacity of the quinoa seed protein concentrate was much higher than that of soybean cake protein concentrate. According to Kamani et al. (158), soy protein concentrate minimizes the cooking/frying loss and shrinkage and enhances foaming stability in chicken sausages.

Various trials have evaluated plant protein concentrates in food applications to reduce the oil ratio and improve the end product's industrial attributes. Plant-based protein hydrolysates have been given significant attention, particularly for enhancing the water-holding ability of meat products (161), which plays a critical role in defining their juiciness, an expression that also refers to the flavor, texture, and color required throughout technological operations (162). In this regard, Carvalho et al. (163) stated that soy protein concentrate employed in beef burger formulation significantly improved the patties' water-holding capacity. Additionally, Hidayat et al. (162) found that this capacity ranged from 86 to 89% in beef sausage and was enhanced by replacing beef with texturized vegetable protein (0–40%). This might be attributed to the existence of more water-soluble components than in animal proteins. According to Karami and Akbariadergani (164), canola protein hydrolysates improved the cooking yield by raising the water-retaining capability of the meat product.

Consumers and the food industry are concerned about lipid oxidation, creating unwanted off-flavors, odors, and possibly serious reaction products (165). Suppressing lipid peroxidation in foodstuffs is critical to prevent food deterioration and protect consumers against hazardous diseases. In this regard, antioxidants are utilized to keep food safe by preventing discoloration and the decay caused by oxidation (166, 167). Despite the extensive use of synthetic antioxidants in food production, the consumers' concern around food safety prompted the food industry to seek natural alternatives (168, 169). Antioxidant proteins and peptides can replace artificial antioxidants since they have an equivalent or higher ability to suppress lipid oxidation (170). Carnosine (β-alanyl-L-histidine) and glutathione (γ-Glu-Cys-Gly) are natural antioxidants in muscle tissue. It has been discovered that they can scavenge hydroxyl radicals, quench singlet oxygen, and restrain lipid oxidation (171). The peptide Pro—Ala—Gly—Tyr separated from Amur sturgeon skin gelatin has scavenging abilities against DPPH, ABTS, and hydroxyl radicals, according to Nikoo et al. (172). The peptide reduced lipid oxidation in minced fish at a concentration of 25 ppm, but it was ineffective at greater concentrations. According to Shahidi et al. (173), incorporation of capelin protein hydrolysate at 0.5–3.0% in a beef model decreased the generation of TBARS by 17.7–60.4%. Over 14 days of storage at 4°C, Kittiphattanabawon et al. (174) assessed lipid peroxidation in treated pork containing gelatin hydrolysate of 40% DH, at concentrations of 100, 500, and 1,000 ppm, and BHA (100 ppm). In both the carotene linoleate and treated pork model systems, they found that gelatin hydrolysate at 500 and 1,000 ppm inhibited lipid peroxidation. Bougatef et al. (175) isolated and purified antioxidant peptides from *Sardinella aurita* proteins by enzymatic hydrolysis. These peptides were found to have a high antioxidant potential in meat-based products. Furthermore, the antioxidant activity of peptides isolated from the mushroom *Ganoderma lucidum* was discovered to reduce lipid oxidation without altering the products' consumer desirability qualities. The antioxidant activity of *G. lucidum* was attributed to the polysaccharide–peptide complex, polysaccharides, and phenolics. Nevertheless, the study found that *G. lucidum* peptide (GLP) is the main antioxidant in *G. lucidum*, which may effectively reduce lipid peroxidation in meat goods by scavenging free radicals, chelating metals, and acting as an antioxidant (176). In a linoleic acid model system, gelatin hydrolysates from cobia (*Rachycentron canadum*) skin delayed lipid oxidation. Cobia gelatin hydrolysate at 8 and 10 mg/mL exhibited a higher inhibitory effect on lipid peroxidation than BHA at 10 mg/L (177). In addition, according to Cai et al. (178), peptides gained from grass carp (*Ctenopharyngodon idella*) skin protein hydrolysate significantly prevented peroxidation in a linoleic acid model system. Sivaraman et al. (179) reported that the squid protein hydrolysate generated by papain has a comparable lipid peroxidation inhibitory capacity as ascorbic acid in the sardine ground meat model system. Similarly, zein hydrolysate has been shown to suppress lipid oxidation, diminish hydrogen peroxide and TBARS generation, and considerably increase the oxidative stability of model oils (180). Furthermore, this hydrolysate shows no adverse effects on emulsion quality and could be used as an effective antioxidant in food emulsion (181). Cuttlefish skin gelatin hydrolysates (0.5 mg/g) prevented turkey sausage lipid peroxidation for up to 10 days at 4°C (182).

Proteins derived from dairy sources are, likewise, high in antioxidant peptides, which could be helpful in the preservation of meat. In this respect, casein calcium peptide (2.0%) combined with beef paste homogenate can suppress around 70% of lipid peroxidation of the homogenate, preventing the formation of odors in meat products and thus, extending their shelf life (183). Additionally, whey protein peptides have also demonstrated their ability to be utilized as functional components in meat goods. Peña-Ramos and Xiong (184) found that adding 2% whey protein hydrolysates to pig meat in cold storage decreased oxidative deterioration and loss during cooking. From the experience of the authors, there are numerous bioactive peptides available. Nevertheless, adaptability with different foods, gastrointestinal stability, bioavailability, and long-term stability must be investigated before application as functional food additives.

### Pharmaceutical Applications

The use of bioactive peptides for pharmaceutical applications is as interesting as that for food purposes. In this context, bioactive peptides and their by-products have been applied as antidiabetic, anticancer, and anti-inflammatory agents, to name a few. Anti-diabetic hydrolysates, for example, can be added to sausages to fortify the sausages with anti-diabetic peptides to reduce the probability of developing diabetes (185). The identity of 24, 30, and 38 bioactive peptides were established in each of three infant milk formulas after separating and identifying bioactive peptides in three hypoallergenic formulas. A large number of these peptides has been identified as ACE inhibitors. The presence of sequences with antihypertensive, hypocholesterolemic, immunomodulation, antibacterial, cytotoxicity, antigenic, antioxidant, and antigenic activities was also established (186). Chou et al. (45) investigated the impact of antioxidant peptides from the chicken liver after enzyme digestion by pepsin and the induction of CAT, GPx, and SOD in D-galactose-induced rats. Comparing the control and the D-galactose-induced groups of rats, the doses of chicken liver hydrolysate administered (0.25 and 0.5 g/kg) resulted in equal or enhanced antioxidant capacity in the liver, heart, kidney, and brain. The researchers discovered that dosages of 0.25 and 0.5 g/kg inhibited the same rate of lipid oxidation in serum and liver as in the control group. Similar findings were also observed by other scientists in terms of the antioxidant potential (*in vivo*) of loach meat hydrolysates (187), chicken breast hydrolysates (32), rice proteins (188), and tilapia collagen (189). Fazhi et al. (68) reported that three peptides (tri-, tetra-, and hexapeptide) were isolated from fermented sesame meal. They found that MDA buildup in serum and liver was decreased by supplementation with any peptide at 0.1, 0.2, or 0.4 g/kg. In addition, all treated mice had higher levels of SOD and GPx.

Numerous bioactive peptides from food have been shown to possess cytomodulatory properties. In particular, peptides recovered from waste whey of mozzarella cheese exhibited an antiproliferative action when evaluated in a human colorectal cancer cell line (190). Similarly, cytomodulatory peptides decreased the growth of cancer cells while also increasing the activity of immune and neonatal intestinal cells (191). The cytotoxic effects of several black cumin extracts as an additional remedy to doxorubicin treatment in human MCF-7 breast cancer cells were also investigated in terms of their anticancer activity. The LC50 of black cumin lipid extract was 2.720 0.2 mg/mL, indicating cytotoxicity. The cytotoxicity of the aqueous extract was evident when the level was as high as 50 mg/mL (192). Furthermore, Saisavoey et al. (193) studied rice bran protein hydrolysate antioxidant and anti-inflammatory properties on the RAW264.7 macrophage cell line, where LPS and rmIFN-g were found to co-stimulate the target protein's inhibitory effect against nitric oxide production. In addition, casein has been discovered to be an abundant source of active opioid peptides. Different casein fragments are hydrolyzed by distinct digestive enzymes, resulting in the formation of peptides with opioid activity (194). These opioid casein fractions were solely discovered in the plasma of newborns, which was surprising. In both animal and human trials, a marketable, valuable 1-casein-derived peptide frequently utilized in confections and soft beverages were shown to have anxiolytic-like stress-relieving characteristics (194).

Plant-based proteins have proven to be a precious source of innovative and effective antihypertensive peptides (113). In this respect, four angiotensin-converting enzyme inhibitory peptides (Val- Trp, Val-Trp-Ile-Ser, Ile-Tyr, and Arg-Ile-Tyr) were identified from rapeseed proteins digested with subtilisin. When orally administered, these peptides were reported to reduce blood pressure in hypertensive rats, with the most significant effect occurring between 2 and 4 h from administration (195). Incorporating these and other antihypertensive peptides into pharmaceutical medicines and functional diets may effectively prevent and treat hypertension. In mammals, antihypertensive peptides also aid in regulating salt balance and fluids (196). Milk-based bioactive peptides might be used to reduce the risk of metabolic syndrome by modulating blood pressure, food consumption, and free radical absorption (197).

### Cosmeceutical Applications

Since a scientific demonstration of the stated bioactivity of novel cosmeceutical substances is frequently required, research in the cosmeceutical sector, which combines cosmetics and pharmaceuticals, is continuously growing. Indeed, one feature that distinguishes cosmeceuticals from traditional cosmetics is the discovery and characterization of active substances and the demonstration of their efficacy in the stated activity (198). Peptides are an important collection of bioactive cosmeceutical components that, due to their unique qualities, suit the majority of the cosmeceutical industry's needs when creating new compositions. In this respect, in addition to bioactivity, two other features of peptides as cosmeceutical components have lately been considered: bioavailability and stability. Moreover, peptides are recognized as valuable cosmetic materials, as they are light and air-stable, present low toxicity, show a powerful affinity for water, and possess moisturizing capabilities (199, 200). Peptides are frequently employed as ingredients in functional cosmetics to treat skin conditions, promoting collagen synthesis and antioxidant, anti-inflammatory, anti-wrinkle, whitening, and wound healing properties (201, 202). Developing new natural peptides and more stable and effective synthetic peptides has sparked renewed interest in peptide-based skincare products (203). Peptides are used as anti-aging skincare due to their ability to synthesize extracellular matrix (ECM) tissue, the disruption of which is key to skin aging (204). Signal, carrier, neurotransmitter inhibitor, and enzyme inhibitor peptides can be categorized as topical cosmeceuticals. Larger molecules can penetrate the skin barrier, particularly in dry and aged skin (205). Synthetic peptides are made up of amino acid chains that may be altered for various purposes, including improved skin penetration, particular receptor binding, stability, and solubility. Finkley and co-authors (206) reported that the facial creams containing GHK-Cu (copper tripeptide 1) applied for 12 weeks on 71 volunteers aged 50–59 resulted in a visible reduction of the signs of aging. In a separate investigation, the same authors tested the formulation on the eyes of 41 pairs of volunteers under comparable experimental conditions, where a cream with vitamin K was used as a control. The cream with GHK-Cu was found to enhance the suppleness and tightness of the skin in both experiments and lessened the appearance of both fine lines and deep wrinkles. Lintner and Peschard (207) found a significant variation in skin permeability amongst palmitoylated and non-palmitoylated peptides. The anti-wrinkle and wound-healing effects of the peptides pal-GHK and pal-AH were examined. The transcutaneous flow was disclosed using standard Franz diffusion cells, which showed increased interpenetration in the case of the palmitoylated analog. The collagen-derived pentapeptide KTTKS is another key peptide active component in cosmeceutical formulations (208). In a fascinating clinical investigation, its palmitoylated analog (pal-KTTKS) was tested and compared to the KTTKS peptide regarding stability and permeability. It was discovered that pal-KTTKS could penetrate all three layers of the skin (stratum corneum, epidermis, and dermis), while unmodified KTTKS was not found in any of them (209). According to previous research, collagen may liberate bioactive peptides with various physiological activities after enzymatic digestion. Collagen peptides/hydrolysates have been found to help improve skin problems (210, 211). Kang et al. (212) employed hairless mice that had been exposed to UV radiation, which were administered 1,000 mg/kg collagen peptide for 9 weeks. Collagen peptides were found to upregulate the expression of hyaluronic acid synthase mRNA and the skin moisturizing factor filaggrin, boost hyaluronic acid concentration in skin tissue, and down-regulate the expression of hyaluronidase (HYAL-1 and HYAL-2) mRNA. Likewise, collagen peptide consumption may prevent skin moisture loss caused by ultraviolet (UVB) light (213). Overall, collagen and synthetic peptides have been widely used to develop anti-aging products and nutraceuticals.

### Wound Healing Applications

Human skin wounds continue to be a substantial and growing public health and economic issue (214). The skin is the largest organ of the human body and serves as a physical barrier between the internal and external environments. Undoubtedly, skin wounds occur frequently in unfortunate accidents. When the skin defenses against hazardous stimuli are compromised, adverse outcomes such as infection, shock, and even death can occur (215, 216). The wound healing process can be slowed down in specific diseases (e.g., diabetes and infection), usually causing chronic wounds (217). Traditional wound healing medications, such as growth factors, cytokines, chemical compounds extracted or produced from plants, and other immunomodulatory agents, have proven to be especially challenging to translate into clinic treatments for chronic wound healing (218).

Bioactive peptides with high activity, specificity, and stability have sparked substantial interest in the associated field of study (219) compared with expensive pharmaceuticals and low activity, safety, and delivery issues. In this regard, in diabetic-ob/ob rats (mutant obese rats employed as animal models of type II diabetes), Carretero et al. (220) found that *in vivo* adenoviral delivery of LL-37 antimicrobial peptides to excisional wounds increased re-epithelialization and granulation tissue formation. Ramos et al. (221) also verified this, finding that LL-37 and PLL-37 (LL-37 derivative containing an N-terminal proline) improve re-epithelialization and angiogenesis in skin lesions with poor wound healing *in vitro* and *in vivo*. Song et al. (222) used electrospun silk fibroin nanofiber membranes to immobilize an LL-37 derivative, Cys-KR12. Cys-KR12 was chosen for its antibacterial and anti-biofilm properties vs. four different bacterial strains (*S. aureus, S. epidermidis, E. coli*, and *P. aeruginosa*) and contained residues 18–29 of the LL-37 sequence. The peptide-modified membranes were discovered to stimulate the proliferation of keratinocytes, fibroblasts, and monocytes, all of which are key to wound healing.

Moreover, collagen peptides serve as fake collagen breakdown peptides in the skin, causing fibroblast cells to create novel collagen fibers in response to a false signal. Collagen peptides also have chemotactic qualities, encouraging cell migration and proliferation, essential to wound healing (223). Recently, marine organisms like fish, fish waste, starfish, sponges, and jellyfish have been investigated as reliable sources of collagen (224). Cheng et al. (225) lately discovered that collagen sponges generated from *Rhopilema esculentum* show potential hemostatic properties, implying that they could be a viable choice for wound treatment. Other biomaterials acquired from marine collagen, such as collagen gels, films, and membranes, have also shown practical applications in wound treatment (226).

### Feed Applications

Enhancing feed utilization efficiency for milk, meat, and egg production is an important goal for animal agriculture. A proper nutrition strategy is required to digest and absorb dietary nutrients in the small intestine. Recently, peptides in animal feeding have received considerable attention (227, 228). Before feeding, chemical, enzymatic, or microbiological procedures are utilized to routinely generate peptides from animal and plant proteins to increase the nutritional quality and decrease any associated anti-nutritional effects (229). After consumption, the proteins in the feed are digested in the small intestine by enzymes and oligopeptidases into small peptides (di- and tri-peptides) and free amino acids (230). Nonetheless, depending on the physiological state of the animals and the composition of their meals, the types of peptides produced might vary substantially. To produce peptides for animal nutrition, only animal by-products, brewer by-products, and plant materials with anti-nutritional elements are hydrolyzed (231). Different peptide compounds have been added to the meals of calves (232), poultry (233), fish (234), and companion animals (235) to enhance their nutrition, gut function, and capacity to combat infectious diseases. According to Kim (236), fermented soybean meal (4.9%) might substitute 3.7% of spray-dried plasma protein in the diet of 3- to-7-week-old pigs given a corn and soybean meal-based diet with no effect on growth performance or feed efficiency. Comparable outcomes were gained for the Atlantic salmon fed a diet including 40% of protein from fermented soy white flakes (233). In the diet of juvenile red sea bream, 50% of the fish meal could be substituted with the equivalent quantity of soybean protein hydrolysate (234). As the fish meal is becoming limited worldwide, adding plant-based protein hydrolysate in diets is critical in aquaculture. Moreover, the hydrolysate of soy protein concentrate (19.7% in diet) can be employed to maintain a sturdy growth in calves as an alternative for expensive skim milk powder (230). In another study, El-Ayek et al. (237) found that black cumin cake can cost-effectively substitute 50% of the protein in forage formulations. El-Deek et al. (238) reported comparable results, confirming that up to 50% black seed cake protein may be used in broiler chick feed with no adverse effects on growth, meat quality, feed consumption, conversion rate, or safety.

### Safety of Bioactive Peptides

Bioactive peptide safety is a significant perspective for clinical studies and food applications. The physiological impact of bioactive peptide consumption (from food and hydrolysate/concentrated forms) is thought to be harmless. Nevertheless, because most toxicological investigations are conducted *in vitro* and in animals, the level of proof supporting the safety of bioactive intake must be increased. To date, just a few investigations on the potential toxicological impact on humans have been undertaken. In this context, according to an *in vitro* work conducted by Doorten et al. (239), daily ingestion of a hydrolysate derived from cow milk (2 g/kg body weight) was not likely to generate mutagenic or clastogenic effects. The scholars found a No Observed Adverse Effect Level (NOAEL) of 40 g/kg body weight/day, 140 times greater than the recommended daily intake. Moreover, Anadón et al. (240) found that acute (2,000 mg/kg) and daily (1,000 mg/kg for 4 weeks) ingestion of casein hydrolysate (rich in antihypertensive peptides) neither had any histological impact nor caused mortality in mice. Overall, peptides are more reactive than natural proteins due to their lower molecular weight and are made up of smaller chains of amino acids. As a result, it is critical to ensure their safety, which includes the absence of toxicity, cytotoxicity, and allergenicity (6). Strict and precise legislation is essential to safeguard consumers from potentially hazardous or deceptive products.

### Peptide Therapeutics Market

Therapeutic peptides and proteins have risen as potential drug candidates for several decades. The peptide therapeutics market is moderately competitive and consists of several major parties. Some companies, which are currently overlooking the market, are Eli Lilly and Company, Pfizer, Inc., Amgen, Inc., Bristol-Myers Squibb Company, EVER NEURO PHARMA GMBH, Takeda Pharmaceutical Company Limited, Davisco Foods International, Tokiwa Yakuhin Co., Ltd., Reliv, Inc., Valio Ltd., and many others. The major partakers are involved in strategic alliances, such as acquisitions and collaborations, along with research activities for the global expansion of the product portfolio. For example, in June 2019, Eli Lilly and Company received the FDA approval for Emgality, a subcutaneously injected calcitonin gene-related peptide (CGRP) antibody, for migraine prevention and treating episodic cluster headache ^1^.

## Conclusion and Future Perspectives

The advantages and activities of bioactive peptides derived from various sources were addressed in this review. Peptide extraction, purification, and identification were also covered. Bioactive proteins can be utilized to develop functional foods and are likely to be employed as a food additive in fatty products to extend their shelf life by increasing oxidative stability. New bioactive peptides derived from various food sources and their by-products for food, pharmaceutical, cosmetic, wound healing, feed, and safety were also discussed. Even though much is known about the structure and activity of peptides, more research into the link between these two aspects is required. Further investigation is needed on the stability of peptide activity and its regulatory factors, in addition to the extraction of bioactive peptides and qualification of prospective bioactivity. In addition, pre-clinical and clinical studies are needed to determine which levels are beneficial for health, their dose-response relation, bioavailability, pharmacokinetics, and whether they can be consumed with foods.

## Author Contributions

AZ and AA contributed significantly to analysis and manuscript preparation. AZ supported valuable discussion. JS-G, J-BE, and J-HS revised the whole manuscript. All authors collated papers, wrote the manuscript, read, and approved the final manuscript.

## Conflict of Interest

The authors declare that the research was conducted in the absence of any commercial or financial relationships that could be construed as a potential conflict of interest.

## Publisher's Note

All claims expressed in this article are solely those of the authors and do not necessarily represent those of their affiliated organizations, or those of the publisher, the editors and the reviewers. Any product that may be evaluated in this article, or claim that may be made by its manufacturer, is not guaranteed or endorsed by the publisher.

## Footnotes

^1^https://www.researchandmarkets.com/reports/5265155/peptidetherapeutics-market-growth-trends. Last access: Dec 19, 2021.
